# RANDOMIZED CLINICAL TRIAL OF ASPIRIN AS PROPHYLAXIS FOR THROMBOEMBOLISM IN HIP ARTHROPLASTY

**DOI:** 10.1590/1413-785220243201e272229

**Published:** 2024-03-22

**Authors:** Raul Carneiro Lins, Epitacio Rolim, Yago Andrade Lima, Rodrigo Rodrigues de Sousa Moura

**Affiliations:** 1 Universidade Federal de Pernambuco, Department of Surgery, Recife, PE, Brazil.; 2 Universidade Federal de Pernambuco, Orthopedics Service at Hospital das Clinicas, Recife, PE, Brazil.

**Keywords:** Disease Prevention, Thromboembolism, Total Hip Replacement, Aspirin, Prevenção de Doenças, Tromboembolia, Artroplastia Total do Quadril, Aspirina

## Abstract

**Objective:**

This study aims to evaluate aspirin as a chemical prophylaxis (200 mg) in total hip arthroplasty.

**Methods:**

the study compared two groups and used ultrasonography (USG) to screen for low-deep venous thrombosis. Group 1 received 600 mg (control), and Group 2 received 200 mg of (intervention), associated with the use of elastic compression stockings and early walking

**Results:**

fourteen patients were allocated to Group A (200mg), and 16 to Group B (600mg); in group A (200mg), 3 cases with thrombus below the popliteal vein were detected at the first USG examination. All of them are in the left lower limb (21.4%). In group B (600 mg), 5 cases were identified after the first exam (31.2%). All cases were asymptomatic and followed the protocol with prophylaxis only with Aspirin.

**Conclusion:**

In the statistical data, there were no differences in the presence of thrombus between the 200- and 600 mg groups, which is credited to using low-dose aspirin in low doses (200mg). Hematimetric levels returned to baseline levels and suggested there was no chronic or acute bleeding related to the use of aspirin. The manuscript was prepared according to the CONSORT guideline 2010. ***Level of Evidence I; Longitudinal Randomized Comparative Clinical Study***.

## RESUMO

**Objetivo:**

Este estudo pretende avaliar a aspirina como profilaxia química (200 mg) na artroplastia total do quadril.

**Métodos:**

estudo comparando dois grupos com diferentes doses de aspirina e utilizando a ultrassonografia (USG) para rastreamento da trombose venosa profunda baixa. O grupo 1, 650 mg ao dia de aspirina (controle) e o grupo 2, 200 mg de aspirina ao dia na mesma posologia (intervenção) e associados ao uso de meias elásticas de compressão e deambulação precoce.

**Resultados:**

quatorze pacientes foram alocados no grupo A (200 mg) e 16 no grupo B (650 mg). No grupo A foram detectados 3 casos com trombos abaixo da veia poplítea ao USG sendo 21,4%. Já no grupo B, 5 casos foram identificados após o primeiro exame (31,2%). Todos assintomáticos e sem sinais de sangramento ativo ou queda da hematimetria no momento da detecção dos trombos.

**Conclusão:**

os dados sugerem não haver diferença na incidência de trombo em ambos os grupos, não sendo a profilaxia com a aspirina dose-dependente. Os níveis hematimétricos retornaram aos níveis iniciais o que sugere não ter havido sangramento crônico ou agudo relacionado ao uso. ***Nível de Evidência I; Estudo Clínico Randomizado Longitudinal Comparativo***.

## INTRODUCTION

Knee and hip joint reconstruction increase exponentially each year. Joint replacement gives a great improvement in life quality. The level of activity for elderly patients and the improvement in the quality of implants are factors that explain the high demand.^1,2^ In the United States, more than 600,000 arthroplasties are performed per year.^3^

Among the complications of joint replacements, infections and thromboembolic events are the most feared. The presence of assimptomatic thrombus in vessels distal to the popliteal vein is estimated between 20 and 30%; however, there´s no need treatment and prophylaxis must be maintained.^1,3,4-5^ Simptomatic deep venous thrombosis may reach an incidence close to 5% and pulmonary thromboembolism, up to 2% in such patients.^7-9^ he ideal drug for the prophylaxis of thromboembolic diseases needs to be highly effective, accessible in dosage and low cost with low risk of postoperative bleeding. There is still no consensus regarding the drug or their ideal dose, and protocols vary between services. Until 2001, when routine prophylactic chemical measures were instituted for arthroplasties, the incidence of deep venous thrombosis and thromboembolic diseases could reach up to 30%.^1,3,7^ Currently, the use of prophylactic measures alone or in association has reduced the incidence close to 2%.

Prophylaxis methods can be divided into chemical or mechanical. Chemical prophylaxis such as warfarin, aspirin, low molecular weight heparin, factor Xa inhibitors and can be used alone or in association with mechanical prophylaxis through the use of pneumatic compression systems in the immediate postoperative period, as well as the early walking in the first 24 hours.^2,6,9^ On the other hand, the use of such substances can cause bleeding of variable magnitude in the postoperative period, requiring a new surgical approach and, therefore, the dose to be administered also becomes a concern for surgeons.^4,6^

Another issue with no consensus is the period of use of chemical prophylaxis which, according to the protocols, can be from 3 to 35 days or even just for the period of hospitalization. In a meta-analysis published in 2020, Gulraj et al. evaluated 13 randomized clinical studies on the use of aspirin associated or not with other drugs, all with different protocols of drugs and doses.^9^ The time of appearance of signs and symptoms of deep venous thrombosis or pulmonary thromboembolism ranges from 21 to 34 days postoperatively.^4,5^ The ideal prophylaxis method should have a low incidence of thromboembolism, as well as postoperative bleeding, and be used for a short period of time.

## METHODS

The study was carried out with 30 patients, all operated on at the orthopedics service of Hospital das Clínicas from May 2019 to May 2021. Goldman grade I or II, without contraindications to the use of aspirin. Patients classified as medium or high risk for thromboembolic disease were excluded, as well as patients with recent episodes of gastrointestinal bleeding, acute myocardial infarction, use of anti-platelet aggregation agents, hip revision surgeries, previous hematological diseases, chronic use of corticosteroids.

Patients who met the inclusion criteria were operated on by the main researcher, using the same surgical approach. The implant used was a complete metal prosthesis with transoperative use of tranexamic acid. After the procedure, the patients were sent to the post-anesthesia recovery room with guidance on the immediate use of elastic compression stockings and with Aspirin scheduled to start 12 hours after the end of the surgery. Patients were divided into two groups by the assistant researcher and blinded to the main examiner. For group A, dose of 300 mg was given twice a day. The period of use was 30 days. Group B received only 200mg twice a day in two doses for 30 days. The patients were randomly distributed by the assistant researcher in a 1:1 ratio, with identification of the dosage used only at the end of the second USG doppler exam, after the sixth week in the end of the postoperative follow-up.

The patients were discharged from the hospital with orientation to use the medication at a dose of 200 or 600mg per day and, in the first postoperative week, were referred to the radiology outpatient clinic of the same hospital for the first ultrasound examination with Doppler flowmetry of the lower limbs for research purposes: thrombus below the popliteal vein. This examination was carried out by two radiologists participating in the research and in case of asymptomatic thrombus, prophylaxis was maintained for the same period. In the cases of patients identified as having a thrombus and symptomatics or signs suggestive of thromboembolic disease, the therapeutic would be adopted instead of the prophylactic one. The patients were instructed to return for the second USG examination with Doppler flowmetry and a new medical review in the sixth week, under the same criteria previously adopted for the diagnosis and treatment of thromboembolic diseases.

The presence of the thrombus below the popliteal vein confirmed by the 2 radiologists present in the study was analyzed as a dichotomous variable as the primary objective of the study. Hematocrit and hemoglobin data before, immediate and late postoperative, in addition to the symptomatological questioning were considered for the evaluation of bleeding due to aspirin use as a secondary objective of the study.

Data were stored using a Microsoft Excel 2010 spreadsheet and analyzed using STATA/SE 12.0 software. The tests were used with 95% confidence and the results presented in tables with their respective frequencies. Numerical variables are presented with measures of central tendency and dispersion. For quantitative variables, the Kolmogorov-Smirnov test was used. The chi-square test and Fisher’s exact test and Student’s t test were applied to categorical variables for variables with normal distribution. As for the variables that did not comply with the normality tests, they were submitted to the Mann-Whitney test for comparison with groups with normal distribution.

The study was approved by the Research Ethics Committee of the Health Sciences Center (CCS) of the Hospital das Clínicas at UFPE and the National Research Ethics Committee (CONEP) and the Ministry of Health under CAAE number: 66155517.2.0000.5208. Obtaining the term of free and informed consent was carried out by the main researcher, consisting of the steps of resolution 466/12 of the Ministry of Health – Brasil

## RESULTS

From May 2019 to May 2021, a total of 30 patients underwent the total hip arthroplasty procedure, according to the inclusion criteria, 14 in group A (200mg) and 16 in group B (600mg) randomized in a 1:1 ratio and blinded to the main examiner. Nineteen (63.3%) were female and 11 (36.7%) were male (p= 0.389). Table 1 Age ranged between 18 and 71 years old with a mean of 49.2 +_ 12.8. Nineteen (63.3%) surgeries were performed on the right hip and 11 on the left hip (36.7%) with no simultaneous bilateral surgery (p= 0.919). Moore access was performed in all cases and two patients in group B required blood transfusion in the immediate postoperative period (12.5%) due to a drop in blood count below 10g/dl of hemoglobin or 30% of hematocrit. However, there was no statistically significant difference. (p=0.485).

**Table 1:** Gender distribution between groups A (intervention) and B (control).

Variable	Group A	Group B	p -value
Genre	a(%)	a(%)	
Female	10 (71.4)	9 (56.2	0.389*
Male	4 (28.6)	7 (43.8)	

The etiological diagnosis was distributed among mechanical causes with 19 cases (63.3%), six from autoimmune diseases (20%) and 5 cases, idiopathic Aseptic Necrosis of the Femoral Head (16.7%).

For the presence of a thrombus detectable on ultrasound examination with Doppler flowmetry, the patients were divided in groups A and B, with the first examination being performed with a mean time of 7.1 days +_ 1.4 for the first group and 5+_ 1.3 days for group B respectively (p = 0.001), with no statistical difference between the mean time for both. (Table 2)

**Table 2:** Incidence of thrombus identified on usg 1 (4-7 days post op).

USG 1 MID		
	Group A	Group B
Yes	0 (0.0)	2 (12.5) 0.485**
No	14 (100)	14 (87.5)
**USG 1 MIE**		
	**Group A**	**Group B**
Yes	3 (21.4)	3 (18.8) 1,000**
No	11 (78.6)	13 (81.2)

In group A (200mg) 3 assynptomatic cases with thrombus below the popliteal vein were detected at the first USG examination with Dopplerflowmetry, all in the left lower limb (21.4%). In group B (600 mg), 5 aassymptomatic cases were identified after the first exam, 2 in the right lower limb and 3 in the left lower limb (31.2%). All cases were asymptomatic and without signs of active bleeding or drop in blood count at the time of thrombus detection and therefore followed the protocol with prophylaxis only with Aspirin in the same dosages already in use. The presence of a thrombus identified by one examiner was confirmed at the same time in a consecutive examination by the second radiologist.

After the sixth postoperative week, the patients came back for the second USG examination with Doppler, after the 30-day period of medication use had ended. In group A, 3 assymptomatic patients (21.4%) with thrombus below the popliteal vein were identified, 1 in the right lower limb and 2 in the left lower limb. (Table 3) In group B, two assymptomatic patients (12.6%) had a thrombus diagnosis confirmed by two radiologists. No patient had clinical signs or symptoms of thromboembolic disease.

**Table 3:** Incidence of thrombus by groups after USG 2 (6 weeks).

USG 2 MID		
	Group A	Group B
Yes	1 (7.1)	1 (6.3) 1,000**
No	13 (92.9)	15 (93.7)
**USG 2 MIE**		
	**Group A**	**Group B**
Yes	2 (14.3)	1 (6.3) 0.586**
No	12 (85.7)	15 (93.7)

(*) Chi-Square Test

(**) Fisher’s Exact Test.

**Figure 1:**
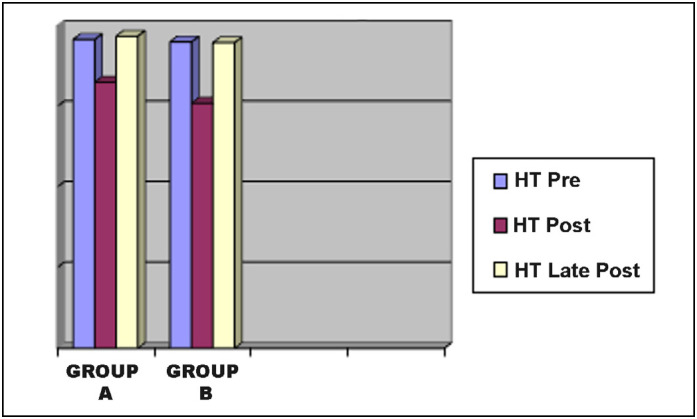
Measured hemoglobin value curve.

**Figure 2:**
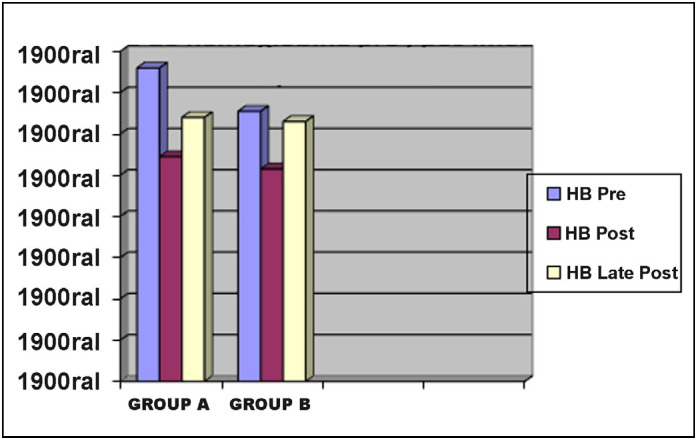
Pre, immediate post and late post hemoglobin curve.

For the hematimetric quantitative variables (hematocrit and hemoglobin), the results were submitted to the Kolmogorov-Smirnov Normality test and Student’s t tests were applied when the normality pattern was observed. Values are shown in graphs 1 and 2 and no significant differences were observed between groups (p> 0.05). (Figures 1 and 2) There were no gastrointestinal complaints of patient-detectable bleeding or surgical wounds in either group.

The relative incidence of thrombi detectable at the first test in both groups (30 patients) was 7 patients and one of them had a positive bilateral test (23.3%). After performing the second imaging exam to detect a thrombus below the popliteal vein, only one new case was diagnosed in group A (3.3%). Two positive exams in the first evaluation were not identified again in the second exam, suggesting thrombus reabsorption. There was no record of bleeding from the surgical wound, gastrointestinal complaint or report of change in stool color suggesting gastrointestinal injury.

Among the 8 patients who presented positive exams, five had rheumatic diseases as etiological diagnosis (62.5%), which suggests, as a post-horc observation, an association between venous thromboembolism and diseases of the autoimmune system.

## Discussion

In 1977 Harris et al. published a prospective study comparing aspirin with placebo in 95 patients undergoing total hip arthroplasty and found an incidence almost 3 times higher in the second group (p< 0.03).^10^ The diagnosis, at the time, was made through contrast radiography of the lower limbs (phlebography). Two years before Zekert et al. identified a significant reduction in necropsy studies of patients who underwent orthopedic surgery and used some drug as prophylaxis of thromboembolic diseases.^11^ Historical studies have already suggested a high incidence of thromboembolic events and the need for chemoprophylaxis in patients undergoing major orthopedic procedures involving the hip joint. However, ther´s no a gold standart fot this solution.

The present study corroborates protocols adopted in some countries such as the United States of America, where the American Association of Orthopedic Surgery (AAOS) (lit), suggests the use of aspirin with level of evidence 1b according to GRADE. In this same guideline developed and published together with the American Academy of Hematology in 2019, the AAOS suggests the association of chemoprophylaxis with mechanical methods such as elastic compression stockings or pneumatic compression pumps for the lower limbs.

In a Chinese study, Zhou et al studied the incidence of venous thromboembolism using Doppler ultrasonography prospectively in a total of 120 randomized patients. There was no statistically significant difference between the aspirin and low molecular weight heparin groups. Both associated with mechanical prophylaxis (p = 0.05).^12^

On the other hand, British works, as well as the National Institute for Health and Care Excellence (NICE), in the last update in 2018, do not recommend aspirin as the drug of first choice for patients undergoing major orthopedic procedures, despite being this recommended conduct with a low level of evidence, with a persistent lack of consensus regarding the ideal drug and duration of use.^13^

In addition, a uniform protocol in centers around the world, the dose of aspirin to be used also becomes a variable without conclusive studies. Parvizi et al demonstrated in a 2021 study the effectiveness of aspirin in major orthopedic surgeries in more than 5000 patients studied at a dose of 325mg twice a day.^14^ On the other hand, when reducing the dose, the same author published a randomized clinical trial showing the efficacy of aspirin at a dose of 81mg a day in 2009.^15^

For the results presented in this study, there was no statistically significant difference between the groups that received doses of 600mg or 200 mg daily for 30 days. None of the patients identified through the USG examination with Doppler developed symptoms due to the presence of the thrombus in veins below the popliteal.

As for the variable presence of clinical signs of gastrointestinal tract bleeding, there was no record of a significant drop in hemocrit or hemoglobin with recovery of preoperative levels after 6 weeks of study, even with the use of aspirin. Some studies show an increased incidence of gastrointestinal bleeding, such as that by Arboy et al. Published in 2020 showing a slightly higher relative risk in patients using aspirin.^16^

Obviously there is a bias in the number of cases with 30 patients, requiring larger and multicenter studies to establish a standard protocol. The COVID-19 pandemic greatly slowed the progression of the study by suspending surgical procedures. The post horc observation of the high incidence of thrombus detectable by USG in patients with rheumatological diseases draws attention to the association between this variable and the non-use of aspirin for these patients. However, studies need to be directed in this direction in order to raise evidence on the subject.
